# Demography and health of Pugs under primary veterinary care in England

**DOI:** 10.1186/s40575-016-0035-z

**Published:** 2016-06-10

**Authors:** Dan G. O’Neill, Elisabeth C. Darwent, David B. Church, Dave C. Brodbelt

**Affiliations:** Production and Population Health, The Royal Veterinary College, Hawkshead Lane, North Mymms, Hatfield, Herts AL9 7TA UK; Clinical Sciences and Services, The Royal Veterinary College, Hawkshead Lane, North Mymms, Hatfield, Herts AL9 7TA UK

**Keywords:** Pug, VetCompass, Electronic patient record, Breed, Dog, Epidemiology, Primary-care, Veterinary, Demography

## Abstract

**Background:**

The Pug is an ancient dog breed and was the fifth most commonly registered UK pedigree breed in 2014. However, the breed has been reported to be predisposed to several disorders including ocular, respiratory and dermatological problems. The VetCompass Programme collates de-identified clinical data from primary-care veterinary practices in the UK for epidemiological research. Using VetCompass clinical data, this study aimed to characterise the demography and common disorders of the general population of Pugs under veterinary care in England.

**Results:**

Pugs comprised 2709 (1.03 %) of 264,260 study dogs under veterinary care from September 1^st^, 2009 to 30^th^ April, 2015. Annual proportional birth rates showed that Pugs rose from less than 1 % of annual birth cohorts before 2008 to comprise 2.8 % of the 2013 annual birth cohort. The most common colours of Pugs were fawn (63.1 %), black (27.7 %), apricot (7.6 %) and silver (2.1 %).

Of the 1009 pugs under veterinary care in the study during 2013, 688 (68.19 %) had at least one disorder recorded. The most prevalent disorders recorded overall were overweight/obesity (number of events: 133, prevalence: 13.18 %, 95 % CI: 11.12–15.43), corneal disorder (88, 8.72 %, 95 % CI: 7.05–10.63) and otitis externa (76, 7.53 %, 95 % CI: 5.98–9.34). The most prevalent disorder groups were ophthalmological (*n* = 164, prevalence: 16.25 %, 95 % CI: 14.03–18.68), dermatological (157, 15.60 %, 95 % CI: 13.38–17.95) and aural (152, 15.06 %, 95 % CI: 12.91–17.42). The most prevalent body locations affected were the head-and-neck (*n* = 439, prevalence = 43.51 %, 95 % CI: 40.42–46.63) and abdomen (195, 19.33 %, 95 % CI: 16.93–21.90). The most prevalent organ systems affected were the integument (321, 31.81 %, 95 % CI: 28.15–35.72) and digestive (257, 25.47 %, 95 % CI: 22.54–28.65). The most prevalent pathophysiologic processes recorded were inflammation (386, 38.26 %, 95 % CI: 34.39–42.27) and congenital/developmental (153, 15.16 %, 95 % CI: 12.61–18.13).

**Conclusions:**

Ownership of Pugs in England is rising steeply. Overweight/obesity, corneal disorder and otitis externa are the most common disorders in Pugs. Identification of health priorities based on VetComapss data can support evidence–based reforms to improve health and welfare within the breed.

**Electronic supplementary material:**

The online version of this article (doi:10.1186/s40575-016-0035-z) contains supplementary material, which is available to authorized users.

## Plain English Summary

The Pug is an ancient breed of Chinese origin that came to England with Prince William of Orange when he gained power as King of England in 1688. The English Stud Book records Pugs as far back as 1859 and Britain’s first Pug Dog Club was approved by the Kennel Club in January 1883. The Kennel Club currently allows four colourings: fawn, black, silver and apricot.

Pugs are currently very popular in the UK and were the fifth most commonly registered UK pedigree dog breed in 2014. Behaviours such as attention seeking and begging for food, and childlike facial features such as large dark round eyes and flat faces are seen as endearing by human owners. However, despite their popularity, the breed has some well-documented health issues, especially in relation to eye, breathing and obesity problems. Collection of health information on large numbers of Pugs attending veterinary practices in England would provide reliable data to assist with reforms that aim to improve the health of the breed. The VetCompass Programme collects de-identified clinical record data from veterinary practices in the UK for research to improve animal welfare.

Pugs made up 2709 (1.03 %) of the 264,260 study dogs under veterinary care from September 1^st^, 2009 to 30^th^ April, 2015. By calculating the proportion of dogs born each year that were Pugs, the study showed that Pugs accounted for less than 1 % of puppies born each year before 2008 annual but rose to comprise 2.8 % of all puppies born in 2013. The most common colours of the study Pugs were fawn (63.1 %), black (27.7 %), apricot (7.6 %) and silver (2.1 %).

Of the 1009 pugs under veterinary care during 2013, 688 (68.19 %) had at least one disorder recorded. The most common disorders recorded were overweight/obesity (13.18 %), corneal (eye surface) problems (8.72 %) and ear infections (7.53 %). The most commonly affected parts of the body affected were the head-and-neck (43.51 %) and abdomen (19.33 %).

This study of over one thousand Pugs provides a framework to identify the most important health priorities in Pugs and can assist with reforms to improve health and welfare within the breed.

## Background

**Fig. 1:**
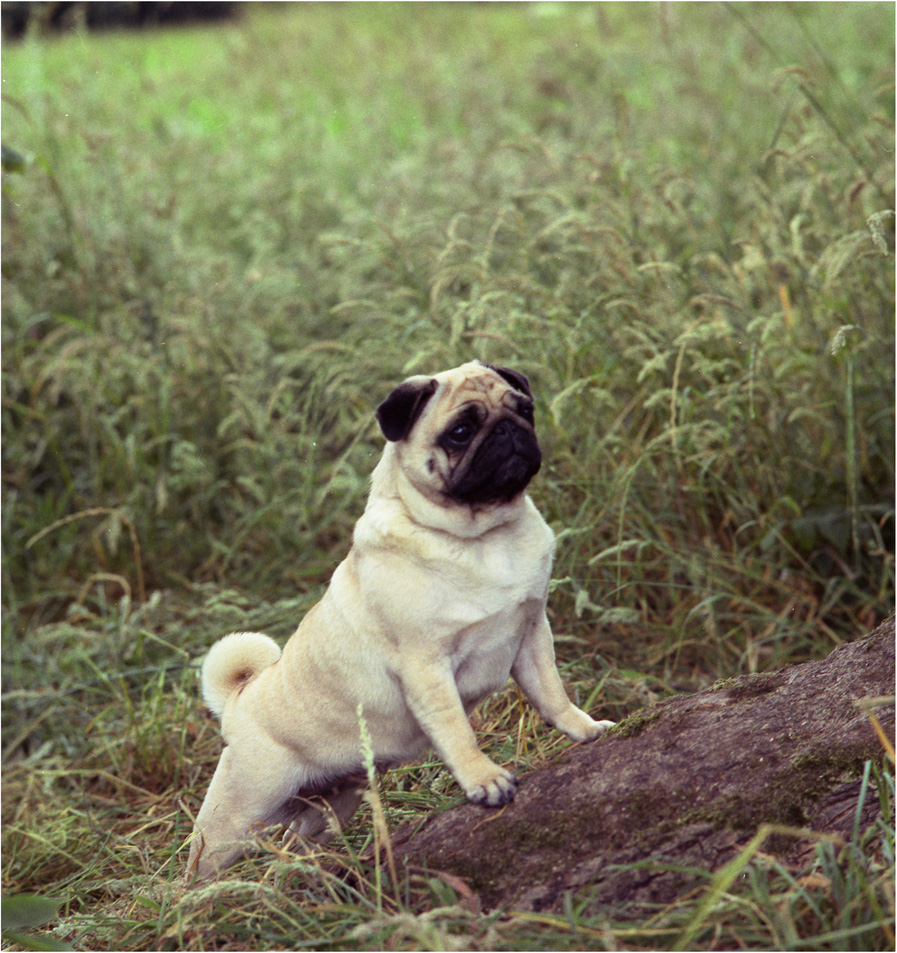
Photograph of a Pug (The Kennel Club©)

The Pug (Fig. 1) is a breed of Chinese origin and is thought to be one of the oldest dog breeds, with references to ‘short-mouthed’ Pug-type dogs documented by Confucius as early as 551 BC [1–3]. The Pug name is thought to derive from the Latin word *pugnus* meaning *fist,* because the side profile of the Pug’s head resembled the shape of a closed fist [1, 2]. The English Stud Book records Pugs as far back as 1859 and the Kennel Club currently allows four colourings: fawn, black, silver and apricot [2, 4, 5] Annual registration data from the UK Kennel Club highlight a dramatic increase in Pug registrations over the past decade, rising over four-fold from 2,116 registrations in 2005 to 9245 registrations in 2014. This makes the Pug the fifth most commonly registered UK pedigree breed for 2014 after the Labrador Retriever, Cocker Spaniel, English Springer Spaniel and French Bulldog [6]. However, despite their popularity, some well-documented health problems have been reported in Pugs [7], many of which paradoxically may be associated with conformational characteristics that also make the Pug fashionable as a breed such as wide, prominent eyes and short muzzles [8–10]. Pugs recorded the second highest count of disorders resulting directly from selection for conformational traits among the top fifty Kennel Club-registered breeds [11] and predispositions to 25 disorders have been reported in Pugs, with ocular, respiratory and dermatological problems especially highlighted [7]. Even among brachycephalic breeds, Pugs appear particularly predisposed to brachycephalic obstructive airway syndrome (BOAS), upper respiratory tract disorders and corneal disorders [8, 12–14]. In recognition of these breed challenges, the UK Kennel Club has listed the Pug as a category 3 breed (the highest category) in its ‘Breed Watch’ system which aims to identify points of concern for individual breeds ‘where some dogs have visible conditions or exaggerations that can cause pain or discomfort’. Points of concern in Pugs highlighted for special attention by show judges include ‘difficulty breathing, excessive nasal folds, excessively prominent eyes, incomplete blink, sore eyes due to damage or poor eyelid conformation, signs of dermatitis in skin folds, significantly overweight, and unsound movement’ [4].

Research using veterinary primary-care clinical data benefits from greater generalizability and reduced selection and recall biases compared with other data sources such as referral or pet insurance data [15]. Using veterinary clinical data from the VetCompass Programme at the Royal Veterinary College (RVC) [16], this study aimed to characterise the demography and common disorders of the general population of Pugs under veterinary care in England. Specific objectives included exploration of bodyweight growth patterns and annual proportional birth data for the general Pug population and to estimate the prevalence of common disorders recorded in Pugs. Syndromic analysis of these disorder data would identify the most common body locations, organ systems and pathophysiological processes affected. The results from the current study could provide a reliable framework to assist reforms in breeding practices and ultimately contribute to improved health and welfare of Pugs.

## Methods

The VetCompass Programme [16] collates de-identified electronic patient record (EPR) data from primary-care veterinary practices in the UK for epidemiological research [17]. Collaborating practices were selected by their willingness to participate and their recording of clinical data within an appropriately configured practice management system. Practitioners could record summary diagnosis terms from an embedded VeNom Code list during episodes of care [18]. Information collected included patient demographic (species, breed, date of birth, sex, neuter status, colour, insurance status and bodyweight) and clinical information (free-form text clinical notes, VeNom summary diagnosis terms and treatment, with relevant dates) data fields.

The sampling frame for the current study included all dogs with at least one EPR (clinical note, VeNom summary diagnosis term, bodyweight or treatment) recorded in the VetCompass database during the study period from September 1^st^, 2009 to 30^th^ April, 2015. These dogs were classified as being *under veterinary care during the study period* and comprised the denominator group in the demographic sections of the study. Date data associated with each EPR event (clinical note, VeNom summary diagnosis term, bodyweight or treatment) were extracted and dogs with a) at least one EPR recorded during 2013 or b) at least one EPR recorded both before and after 2013 were accepted as being *under veterinary care during 2013* and were included as the denominator group for the disorder prevalence sections of the study. Ethics approval was granted by the RVC Ethics and Welfare Committee (reference number 2015/T310).

### Demography

Demographic evaluation used data recorded on all dogs in the VetCompass database *under veterinary care during the study period.* Dogs recorded as Pug breed were categorised as Pug and all remaining dogs were categorised as non-Pug. Year of birth from 2003 to 2014 was derived for each dog using the birth dates recorded in the EPRs. Annual proportional birth rates for Pugs described the proportion of Pugs relative to all dogs born in each year from 2003 to 2014. Bodyweight data with their associated dates were used to generate separate bodyweight growth curves for male and female Pugs by plotting age-specific bodyweights and were overlaid with a fractional-polynomial prediction plot using the Stata *fpfit* command [19]. Coat colour data recorded in the EPRs were used to describe the most common colors of Pugs.

### Disorder prevalence

Disorder prevalence evaluation used clinical data recorded on Pugs in the VetCompass database that were *under veterinary care during 2013* to report one-year period prevalence values. *Age* described the age (years) for each Pug at the earlier of either December 31^st^, 2013 or the date of death and was categorised into six groups (<3.0, 3.0–5.9, 6.0–8.9, 9.0–11.9, ≥ 12.0, not recorded). *Bodyweight* described the maximum bodyweight recorded during the study period for mature dogs (older than nine months) and was categorised into seven groups (0.0–4.9 kg, 5.0–6.9 kg, 7.0–8.9 kg, 9.0–10.9 kg, 11.0–12.9, ≥ 13.0 kg, not recorded). N*euter* described the status of the dog (neutered or entire) recorded at the final EPR and i*nsurance* described whether a dog was insured at any point during the study period.

All clinical notes and VeNom summary diagnosis terms recorded from January 1^st^, 2013 to December 31^st^, 2013 for Pugs under veterinary care during 2013 were reviewed in detail and the most definitive diagnostic term recorded for each disorder was manually linked to the most appropriate VeNom term as previously described (manual review by ED and DON) [17]. Elective (e.g., neutering) or prophylactic (e.g., vaccination) clinical events were not included. Multiple counting of disorder events for ongoing cases was avoided by including recurring diagnoses of ongoing conditions only once (e.g., repeated events of otitis externa) and by including only the final diagnosis for cases with diagnostic refinement over time (e.g., following clinical work-up or trial therapy), based on the assumption that diagnostic accuracy increased over time [20]. The parent term alone was used for disorders that encompassed multiple child terms [21] (e.g., a parent term *traumatic injury* may have multiple child terms such as *laceration*, *fracture* and *hypovolaemic shock*). Disorder events that were aetiologically independent despite sharing the same disorder term name (e.g., novel traumatic events) were included separately. No distinction was made between pre-existing and incident disorder presentations. Disorders described within the clinical notes using presenting sign terms (e.g., ‘vomiting’ or 'vomiting and diarrhoea'), but without a formal clinical diagnostic term being recorded, were included using the first sign listed (e.g., vomiting). Dental disorders were included only where clinical management intervention was recommended. Overweight/obesity was recorded for any animals where the clinical notes expressly indicated that the animal was above its optimal weight or a recommendation was recorded that the animal should lose weight.

The VeNom diagnosis terms were mapped to three hierarchy systems for analysis: diagnosis-level precision, grouped-level precision and syndromic classification as previously described [17]. Briefly, diagnosis-level precision terms were one-to-one descriptors of the original extracted terms describing the maximal diagnostic precision recorded within the clinical notes (e.g., *inflammatory bowel disease* would remain as *inflammatory bowel disease*). Grouped-level precision terms were one-to-one descriptors of original diagnosis terms mapped to a general level of diagnostic precision (e.g., *inflammatory bowel disease* would map to *enteropathy*). Syndromic classification mapped the original VeNom diagnosis terms to three taxonomic groupings: body location, organ system and pathophysiologic process, and each original diagnostic term could be mapped to multiple syndromic terms [17]. Full details on the mapping systems used between the diagnosis-level precision, grouped-level precision and syndromic classifications are provided as Additional file 1 to this paper.

Following data checking and cleaning in Excel (Microsoft Office Excel 2013, Microsoft Corp.) to ensure internal data consistency (e.g., that all dogs that died also had a recorded date of death) and data validity [22], analyses were conducted using Stata Version 13 (Stata Corporation). The sex, neuter status, insurance, age and adult bodyweight for Pugs under veterinary care during 2013 were described. A cross-sectional study design was used to estimate a one-year (2013) period prevalence with 95 % confidence intervals (CI) that described the probability for disorders occurring at least once during 2013. The CI estimates were derived from standard errors based on approximation to the normal distribution for disorders with ten or more events [23] or the Wilson approximation method for disorders with fewer than ten events [24]. These methods were applied to report prevalence based on diagnosis-level precision, grouped-level precision and syndromic classifications.

## Results

### Demography

**Fig. 2:**
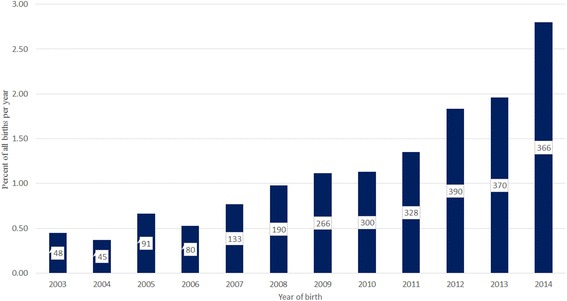
Annual proportional birth rates (2003–2014) for Pugs among all dogs (*n* = 263,456) attending primary-care veterinary clinics in England participating in the VetCompass Programme. The annual birth count of Pugs is shown in each bar

The denominator group used for demographic analysis comprised 264,260 dogs *under veterinary care during the study period* from September 1^st^, 2009 to 30^th^ April, 2015, and included 2709 (1.03 %) Pugs. Of the 263,456 (99.70 % of the sampling frame) dogs with a valid date of birth available, there were 2695 (1.02 %) Pugs. Annual proportional birth rates showed that Pugs rose from less than 1 % of the pre-2008 annual VetCompass birth cohorts to comprise 2.8 % of the annual birth cohort born in 2013 (Fig. 2).

**Fig. 3:**
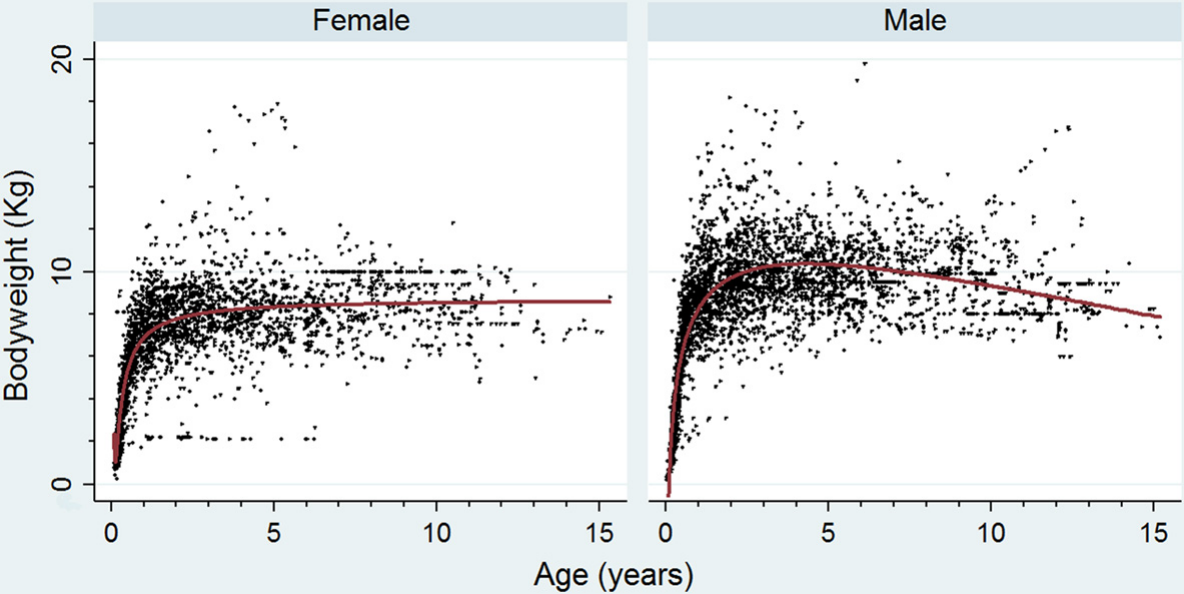
Bodyweight growth curves overlaid with a fractional-polynomial prediction plot for female and male Pugs attending primary-care veterinary clinics in England participating in the VetCompass Programme. (Females *n* = 772, Males *n* = 992)

Bodyweight growth curves were generated using 4075 bodyweight-date values of 922 male Pugs and 3062 bodyweight-date values of 772 female Pugs that had at least one bodyweight value recorded. These bodyweight growth curves showed rapid growth during the first year of life (Fig. 3). The median bodyweight across all ages for males (8.9 kg, IQR: 7.1–10.3, range: 0.2–19.8) was higher than for females was (7.3 kg, IQR: 5.0–8.5, range: 0.3–17.9) (*P* < 0.001).

Coat colour data were available for 2693 of the 2709 Pugs (99.41 %). Of the Pugs with coat colour recorded, the most common colours included fawn (*n* = 1652, 63.1 %), black (747, 27.7 %), apricot (204, 7.6 %) and silver (56, 2.1 %). The remaining 34 (1.3 %) dogs were variously recorded as brown (*n* = 8), white (8), brindle (6), sable (4), chocolate (4), champagne (2) or red (2). Sex data were available on 2678 of the 2709 (98.9 %) Pugs, of which 1259 (47.0 %) were female and 1,419 (53.0 %) were male.

### Common disorders

There were 104,233 dogs *under veterinary care during 2013* that had either at least one EPR recorded from January 1^st^ 2013 to December 31^st^ 2013 (96,319 dogs) or otherwise at least one EPR before and after 2013 (7914 dogs). Of these, there were 1009 (0.97 %) Pugs that comprised the denominator group for disorder prevalence evaluation. These Pugs were registered across 102 veterinary practices, with a median count of seven Pugs per practice (interquartile range [IQR] 4–13, range 1–52). There were 688 (68.19 %) Pugs with at least one disorder recorded during 2013 while the remainder (31.81 %) had no disorder recorded. The median count of disorders per dog during 2013 was one disorder (IQR 0–3, range 0–9).

**Fig. 4:**
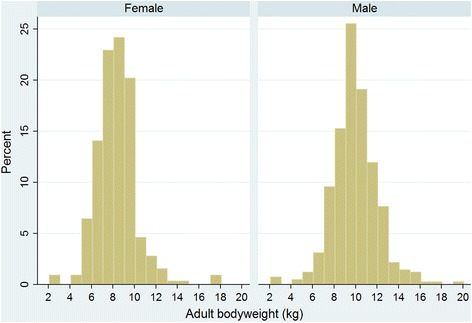
Graphs by Female and Male. Adult (≥9 months) bodyweight of female and male Pugs under veterinary care at practices in England participating in the VetCompass Programme during 2013. (Females *n* = 327, Males *n* = 419)

**Fig. 5:**
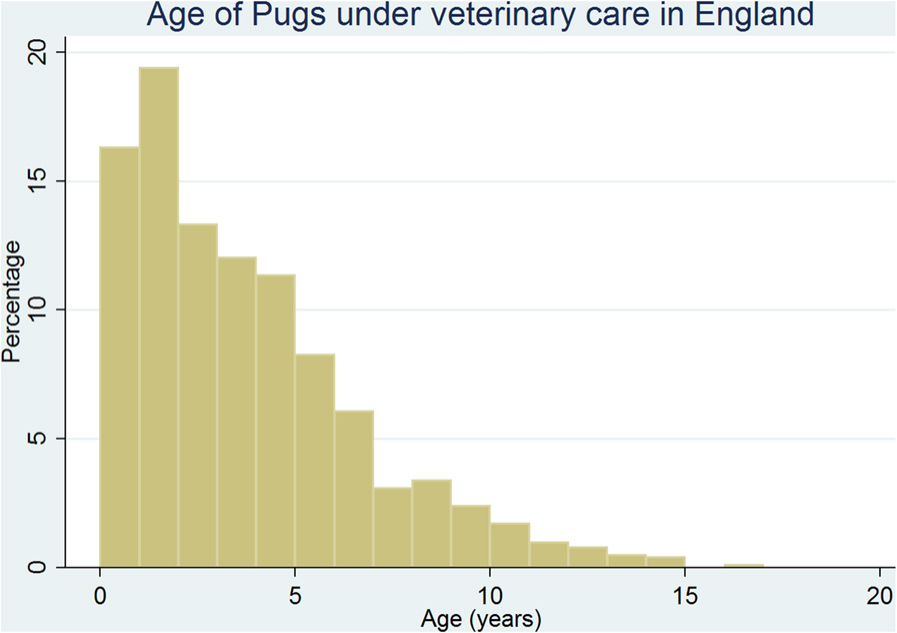
Ages of Pugs under veterinary care at practices in England participating in the VetCompass Programme during 2013 (*n* = 1,006)

Of the Pugs with information available, 455 (45.6 %) were female, 312 (61.1 %) were neutered and 258 (53.3 %) were insured. The median adult bodyweight overall was 9.2 kg (IQR: 7.9–10.4, range: 2.1–19.8) (Table 1). The median adult bodyweight for males (9.9 kg, IQR: 8.8–11.1, range: 2.7–19.8) was higher than for females was (8.2 kg, IQR: 7.3–9.3, range: 2.1–17.9) (*P* < 0.001) (Fig. 4). The median age of the Pugs under veterinary care during 2013 was 3.0 years (IQR: 1.3–5.2 range: 0.0–16.8) years (Fig. 5).

**Table 1:** Demography of Pugs under primary veterinary care in England from January 1^st^, 2013 to December 31^st^, 2013 (*n* = 1009)

		All records		Complete records only	
Variable	Category	No.	Percent	No.	Percent
Sex	Female	455	45.1	455	45.6
	Male	544	53.9	544	54.4
	Not recorded	10	1.0	~	~
Neuter status	Entire	199	19.7	199	38.9
	Neutered	312	30.9	312	61.1
	Not recorded	498	49.4	~	~
Adult bodyweight (aged ≥ 9 months) (kg)	<5.0	11	1.1	11	1.5
	5.0–6.9	72	7.1	72	9.6
	7.0–8.9	254	25.2	254	34.0
	9.0–10.9	276	27.4	276	37.0
	11.0–12.9	104	10.3	104	13.9
	≥13.0	30	3.0	30	4.0
	Not recorded	263	26.0	~	~
Age category (years)	<3.0	493	48.9	493	49.0
	3.0–5.9	318	31.5	318	31.6
	6.0–8.9	126	12.5	126	12.5
	9.0–11.9	51	5.1	51	5.1
	≥12.0	18	1.8	18	1.8
	Not recorded	3	0.3	~	~
Insurance	Not insured	226	22.4	226	46.7
	Insured	258	25.6	258	53.3
	Not recorded	525	52.0	~	~

During 2013, 1641 unique disorder events that encompassed 213 distinct diagnosis-level precision disorders were recorded. The most prevalent diagnosis-level precision disorders recorded were overweight/obesity (number of events: 133, prevalence: 13.18 %, 95 % CI: 11.12–15.43), corneal disorder (88, 8.72 %, 95 % CI: 7.05–10.63), otitis externa (76, 7.53 %, 95 % CI: 5.98–9.34), unspecified ear disorder (75, 7.43 %, 95 % CI: 5.89–9.23), anal sac impaction (66, 6.54 %, 95 % CI: 5.09–8.25) and periodontal disease (62, 6.14 %, 95 % CI: 4.74–7.81) (Table 2)

**Table 2:** Prevalence of the 25 most common disorders at their greatest diagnostic precision recorded in Pugs attending primary-care veterinary practices participating in the VetCompass Programme in England from January 1^st^, 2013 to December 31^st^, 2013 (*n* = 1009)

Diagnosis-level disorder	Disorder count	Prevalence %	95 % CI
Overweight/obesity	133	13.18	11.12–15.43
Corneal disorder	88	8.72	7.05–10.63
Otitis externa	76	7.53	5.98–9.34
Unspecified ear disorder	75	7.43	5.89–9.23
Anal sac impaction	66	6.54	5.09–8.25
Periodontal disease	62	6.14	4.74–7.81
Nails overlong	57	5.65	4.31–7.26
Brachycephalic obstructive airway syndrome (BOAS)	52	5.15	3.87–6.70
Vomiting	50	4.96	3.70–6.48
Diarrhoea	38	3.77	2.69–5.13
Upper respiratory tract noise increased	36	3.57	2.51–4.91
Intertrigo	32	3.17	2.18–4.45
Retained deciduous tooth	31	3.07	2.10–4.33
Umbilical hernia	28	2.78	1.85–3.99
Respiratory noise increased	28	2.78	1.85–3.99
Lameness	24	2.38	1.53–3.52
Ocular discharge	22	2.18	1.37–3.28
Pruritus	22	2.18	1.37–3.28
Pyotraumatic dermatitis	21	2.08	1.29–3.16
Alopecia	20	1.98	1.21–3.04
Conjunctivitis	19	1.88	1.14–2.93
Keratoconjunctivitis sicca	19	1.88	1.14–2.93
Coughing	18	1.78	1.06–2.80
Pyoderma	17	1.68	0.98–2.68

There were 48 distinct grouped-level precision disorders recorded. The most prevalent grouped-level precision disorders were ophthalmological (*n* = 164, prevalence: 16.25 %, 95 % CI: 14.03–18.68), dermatological (157, 15.60 %, 95 % CI: 13.38–17.95), aural (152, 15.06 %, 95 % CI: 12.91–17.42), overweight/obesity (133, 13.18 %, 95 % CI: 11.15–15.43) and enteropathy (113, 11.20 %, 95 % CI: 9.32–13.31) (Table 3).

**Table 3:** Prevalence of the 25 most common groups of disorders recorded in Pugs attending primary-care veterinary practices in England participating in the VetCompass Programme from January 1^st^, 2013 to December 31^st^, 2013 (*n* = 1009)

Grouped-level disorder	Count	Prevalence	95 % CI
Ophthalmological	164	16.25	14.03–18.68
Dermatological	157	15.60	13.38–17.95
Aural	152	15.06	12.91–17.42
Overweight/obesity	133	13.18	11.15–15.43
Enteropathy	113	11.20	9.32–13.31
Upper respiratory tract	105	10.41	8.59–12.46
Congenital	91	9.02	7.32–10.96
Claw/nail	73	7.23	5.71–9.01
Anal sac	70	6.94	5.45–8.68
Dental	66	6.54	5.09–8.25
Musculoskeletal	52	5.15	3.87–6.70
Lower respiratory tract	43	4.26	3.10–5.70
Mass lesion	35	3.47	2.43–4.79
Parasite infestation	35	3.47	2.43–4.79
Female reproductive system	30	2.97	2.01–4.22
Hernia	30	2.97	2.01–4.22
Brain	29	2.87	1.93–4.10
Traumatic injury	27	2.68	1.77–3.87
Urinary system	21	2.08	1.29–3.16
Lethargy	17	1.68	0.98–2.68
Neoplasia	15	1.49	0.83–2.44
Spinal cord	14	1.39	0.76–2.32
Undesirable behavioural	13	1.29	0.69–2.19
Collapse/syncopal episodes	8	0.79	0.40–1.56
Foreign body	8	0.79	0.40–1.56

Syndromic classification analysis indicated that 601 (59.56 %) Pugs had at least one body location affected. The most prevalent body locations affected were the head-and-neck (*n* = 439, prevalence = 43.51 %, 95 % CI: 40.42–46.63), abdomen (195, 19.33 %, 95 % CI: 16.93–21.90), thorax (126, 12.49 %, 95 % CI: 10.51–14.69) and limb (122, 12.09, 95 % CI: 10.14–14.26) (Table 4).

**Table 4:** Prevalence of body locations affected by at least one disorder recorded in Pugs attending primary-care veterinary practices in England participating in the VetCompass Programme from January 1^st^, 2013 to December 31^st^, 2013 (*n* = 1009)

Body location	Count	Prevalence	95 % CI
Head and neck	439	43.51	40.42–46.63
Abdomen	195	19.33	16.93–21.90
Thorax	126	12.49	10.51–14.69
Limb	122	12.09	10.14–14.26
Anus/Perineum	76	7.53	5.98–9.34
Pelvis	15	1.49	0.83–2.44
Vertebral column	10	0.99	0.48–1.82
Tail	1	0.10	0.02–0.56

At least one organ system was affected in 671 (66.50 %) Pugs. The most prevalent organ systems affected were the integument (321, 31.81 %, 95 % CI: 28.15–35.72), digestive (257, 25.47 %, 95 % CI: 22.54–28.65), respiratory (190, 18.83 %, 95 % CI: 15.29–22.97) and connective/soft tissue (181, 17.94 %, 95 % CI: 14.79–21.59) (Table 5).

**Table 5:** Prevalence of organ systems affected by at least one disorder recorded in Pugs attending primary-care veterinary practices in England participating in the VetCompass Programme from January 1^st^, 2013 to December 31^st^, 2013 (*n* = 1009)

Organ system	Count	Prevalence	95 % CI
Integumentary	321	31.81	28.15–35.72
Digestive	257	25.47	22.54–28.65
Respiratory	190	18.83	15.29–22.97
Connective/Soft tissue	181	17.94	14.79–21.59
Ocular	170	16.85	14.22–19.84
Auditory	160	15.86	13.15–19.00
Musculoskeletal	69	6.84	5.44–8.57
Nervous	53	5.25	3.90–7.05
Reproductive	51	5.05	3.71–6.85
Urinary	32	3.17	2.31–4.34
Endocrine	10	0.99	0.54–1.81
Cardiovascular	6	0.59	0.27–1.29
Haematopoietic	6	0.59	0.27–1.29
Hepatobiliary	1	0.10	0.02–0.56
Lymphatic	0	0.00	0.00–0.38

At least one affected pathophysiological process was described in 591 (58.57 %) Pugs. The most prevalent pathophysiologic processes recorded were inflammation (386, 38.26 %, 95 % CI: 34.39–42.27), congenital/developmental (153, 15.16 %, 95 % CI: 12.61–18.13), nutritional (148, 14.67 %, 95 % CI: 11.69–18.24) and hereditary (110, 10.90 %, 95 % CI: 8.70–13.57) (Table 6).

**Table 6:** Prevalence of pathophysiological processes affected by at least one disorder recorded in Pugs attending primary-care veterinary practices in England participating in the VetCompass Programme from January 1^st^, 2013 to December 31^st^, 2013 (*n* = 1009)

Pathophysiological process	Count	Prevalence	95 % CI
Inflamatory	386	38.26	34.39–42.27
Congenital/Developmental	153	15.16	12.61–18.13
Nutritional	148	14.67	11.69–18.24
Hereditary	110	10.90	8.70–13.57
Infectious	103	10.21	8.38–12.38
Mass/Swelling	90	8.92	7.37–10.75
Traumatic	51	5.05	4.03–6.32
Degenerative	36	3.57	2.62–4.84
Parasitic	35	3.47	2.52–4.75
Immune-mediated	30	2.97	2.02–4.35
Allergic	26	2.58	1.83–3.62
Behavioural	15	1.49	0.83–2.66
Iatrogenic	14	1.39	0.80–2.38
Metabolic	14	1.39	0.74–2.58
Neoplastic	13	1.29	0.78–2.13
Foreign body-related	10	0.99	0.58–1.69
Intoxicative	8	0.79	0.40–1.56
Haemostatic	6	0.59	0.27–1.29
Effusion	0	0.00	0.00–0.38
Thermoregulatory	0	0	0.00–0.38

## Discussion

This study of over one thousand animals is the largest analysis of breed health in Pugs based on primary-care veterinary records to date. The results highlight steeply rising Pug ownership in England, with Pugs comprising over 2.5 % of all dogs attending veterinary practices that were born in 2013, although not all of these may have been born in the UK. The most common disorders identified in Pugs were overweight/obesity, corneal disorder and otitis externa and the most commonly affected body location was the head-and-neck region. These results provide a framework to identify health priorities in Pugs that can contribute positively to reforms to improve health and welfare within the breed.

A good understanding of demography is critical for optimal interpretation and generalization of canine health studies [25–27]. The current study identified a steep rise in Pug ownership in recent years. Annual proportional birth rates have risen almost three-fold from less than 1 % before 2008 to almost 3 % of the annual birth cohort born in 2013 (Fig. 2). These findings are consistent with registration data from the UK Kennel Club that recorded an increase of more than four-fold in Pug registrations between 2005 and 2014, [6]. Unfortunately the current study was unable to identify between Kennel Club registered and unregistered dogs but work is underway within the VetCompass Programme to enable such distinction and could contribute to greater clarity on the health comparisons between these two groups. The popularity of the Pug breed has been ascribed to anthropomorphic tendencies of owners who perceived as endearing their child-like or baby-like (paedomorphic) physical characteristics such as flat faces and large eyes [10, 28] and behaviours such as tractability, attention seeking, begging for food and waiting patiently [29–31]. Social effects such as celebrity endorsement and product advertising that features Pugs may also strongly influence breed selection decisions by prospective puppy buyers [32–36].

However, increasing popularity of individual breeds is not necessarily a benign phenomenon. Extreme conformational features of the Pug such as large dark round eyes and flat faces that are appealing to humans have been associated with welfare concerns for the dogs [10, 28, 37]. There are also fears that increased demand for Pugs may contribute to suboptimal breeding and welfare standards as breeders and suppliers rapidly attempt to fulfil the heightened consumer demand [38]. Large numbers of Pug puppies are reported to be imported into the UK, both legally and illegally, with consequent health and behavior risks to these puppies themselves as well as concerns about the introduction of non-endemic diseases such as rabies into the UK [39–41]. Consequently, surveillance of the health of the general population of Pugs in the UK is of increasing importance to support both dog welfare and UK national disease status activities.

Bodyweight growth curves are commonly used for health screening and surveillance in humans, especially because of increasing public health concern over childhood undernutrition and obesity [42] and may similarly contribute to improved nutritional health in dogs. However, diversity between dog breeds in their relative and absolute growth rates implies canine growth curves should be generated from populations that are representative by sex, breed and geography for the target animals for optimal reliability [43]. Unsurprisingly, the results confirmed that male Pugs (median bodyweight 8.9 kg) are heavier than female Pugs (median bodyweight 7.39 kg) and the current study provides exemplars of growth curves for the general population of male and female Pugs in England and could assist breeders and veterinarians to recognise individual animals that are over- or under-sized. Canine growth curves could also assist with estimation of age, for example in puppies presented for importation to the UK to validate the consistency between the stated age and bodyweight [40].

During the one year period of surveillance (2013) of this study, 32 % of Pugs under veterinary care did not have any disorders recorded and were instead either presented for routine or prophylactic veterinary care or did not attend the veterinary clinic at all. This value is higher than the 24 % of dogs without any recorded disorders that was reported across a random sample of all breeds in the VetCompass database [17]. The lower proportion of Pugs recorded with at least one disorder may partially be explained by the younger age of Pugs in the current study (median age: 3.0 years) compared with the the overall dog population in the previous study (median age: 4.5 years). However, these results also suggest that, despite well-documented health concerns in Pugs, many individuals in this breed are not diagnosed with illness over extended periods of observation and that gaining a full understanding of health issues in Pugs will be a complex undertaking that also requires data on disease duration and severity [7, 11].

A review of breed predispositions to disease identified 25 disorders have been reported as over-represented in Pugs [7]. A loose comparison between these breed predispositions and the current study identified just eight of the 25 over-represented disorders that were also among the 25 most common disorders recorded in Pugs shown in Table 2: corneal disorder, brachycephalic obstructive airway syndrome (BOAS), upper respiratory tract noise increased, intertrigo, respiratory noise increased, pruritus, keratoconjunctivitis sicca and coughing. This highlights the importance of generating both ‘between breed’ and ‘within breed’ evidence on disorder occurrence in order to facilitate disorder prioritisation based on both relative and absolute disorder burdens for individual breeds.

Overweight/obesity was the most common single disorder recorded in the current study, with 13 % of Pugs affected. By comparison, just 6 % of dogs across all breeds were recorded with overweight/obesity in a VetCompass study using a similar methodology [17]. Obesity has previously been recognised as a serious concern in Pugs. The Kennel Club cite being significantly overweight as a point of concern for special attention by show judges under its Breedwatch scheme [4]. In a study of show-visiting dogs, Pugs had the second highest mean body condition of 64 dog breeds evaluated [44]. Although the current study concurs with the literature that overweight/obesity is a common finding in Pugs, the true prevalence may be substantially under-estimated in the current retrospective study. Compared with retrospective studies, prospective studies of obesity in dogs generally report much higher overweight/obesity values ranging from 25 % to 41 % [45–47]. Obesity is a clinically relevant disorder in dogs because of associations with disorders including diabetes mellitus, cardiovascular, skin and musculoskeletal disease, exercise and heat intolerance, metabolic syndrome and increased surgical and anaesthetic risk [13, 47–49]. Overweight/obesity should be considered a health priority in Pugs because of the high prevalence, associated health problems and reversible nature of the disorder [50].

Corneal disorders were the second most common specific disorder recorded in the current study, with almost 9 % of Pugs affected during the study year. In contrast, corneal disorders did not rank among the top twenty disorders recorded across all dog breeds in England [17]. Corneal disorders cover a spectrum of presentations including pigmentation, opacity, vascularisation, scarring, erosion, ulceration and perforation, many of which are non-specific biological responses to various noxious stimuli, including mechanical abrasion, immune-mediated keratitis, trauma, and tear film disorders [51]. A high prevalence of primary periocular and ocular problems, including macroblepharon, entropion, distichiasis, ectopic cilia and keratoconjunctivitis sicca, has been previously documented in Pugs, and secondary corneal disease may ensue [52–56]. Selection towards exaggerated brachycephalic facial morphologies have been suggested to promote the occurrence of ocular disorders in Pugs [10, 37, 57, 58]. The UK Kennel Club has taken efforts to redress these associations by listing Pugs as a category 3 breed in their ‘Breed Watch’ system and has recently revised the Pug breed standard to discourage severe exaggeration [4, 8]. Given that the canine cornea is densely innervated by nociceptive afferent axons and that corneal damage is believed to cause substantial pain [59], the results from the current study showing that ophthalmological disorders were the most common grouped disorder in Pugs, with over 16 % of dogs affected, and that the head-and-neck was the most common body location for disorders, with over 40 % of dogs affected, suggest that prioritisation of the extent of brachycephaly for ongoing health surveillance and reform will be important to improve the health welfare of Pugs.

Brachycephalic obstructive airway syndrome (BOAS) defines variable clinical presentations resulting from underlying primary or secondary disorders that include stenotic nares, enlarged tonsils, elongated soft palate, everted lateral saccules of the larynx, narrowed rima glottides, collapse of the larynx and tracheal hypoplasia [60–62]. Dogs affected by BOAS often have severe dyspnoea and inspiratory stridor leading to exercise intolerance and potentially heat stress [62]. Unremitting or remitting breathlessness (‘air hunger’) can have severe welfare implications over prolonged proportions of the lives of affected dogs [61, 63], with severely affected animals being fully engaged in active breathing efforts to the detriment of other life activities [64]. In a prospective study of BOAS in Pugs, 88 % of Pugs attending a referral veterinary hospital and 91 % of a general population of Pugs were diagnosed with BOAS based on clinical history, owner questionnaire and clinical examination [14]. However, the current retrospective study using primary-care veterinary clinical records identified just over 5 % of Pugs that were specifically diagnosed with BOAS and 10 % of Pugs recorded with at least one upper respiratory disorder. These contrasting data suggest that many BOAS-affected Pugs may be accepted as ‘normal for breed’ because of the pervasive true prevalence of the disorder and that only the most severely affected cases may receive a formal BOAS diagnosis [65]. In support of this normalisation phenomenon, more than half of owners of dogs diagnosed with BOAS at a referral centre stated that their dog *did not* have breathing problems [66]. Such normalisation phenomena may blind owners and veterinarians to disorders in commonly affected breeds and constrain reforms intended to improve the welfare of affected breeds [61, 66, 67] as well as also contributing to the apparently low prevalence of BOAS and other respiratory problems recorded in the current study.

Two congenital or early-developmental disorders, retained deciduous teeth (3.0 %) and umbilical hernia (2.8 %), also featured amongst the most common disorders in Pugs. By contrast, neither disorder appeared in the top twenty disorders recorded across all breeds in a previous study [17]. This suggests that retained deciduous teeth and umbilical hernia may be more common in Pugs than in the general population of dogs. However, these findings need to be interpreted carefully because, as discussed above, the relative excess of younger individuals in the current study may positively confound the apparent risks for disorders that occur during the early life period.

The study had some limitations. Studies based on reviews of medical records of animals may under-estimate the true disease burden by predominantly including those more severely affected animals that warrant veterinary management and there may be reduced reporting of less severely affected animals that may be less likely to be clinically presented [61]. The practices participating in the study were situated mainly in central and south-east England and therefore may not be fully representative of the overall veterinary practice structure and caseloads in England. Case definitions and diagnosis recording relied heavily on the clinical acumen and note-making of attending practitioners [17]. The current study ranked disorders based on prevalence but additional data on duration and severity are also required for effective welfare prioritisation of disorders [68, 69]. Clinical variants of some diseases may be recorded using distinct disorder terms and therefore the overall prevalence for these diseases may be fragmented into separate prevalence values for each of multiple more-specific diagnostic term, giving the illusion of lower prevalence [17]. The current study attempted to overcome such artefacts by also providing grouped-level precision results for common disorders to improve interpretability. Reporting the maximum bodyweight recorded during the adult period of life may have upwardly biased the bodyweight results compared with average bodyweights of Pugs at any one point in time. Additionally, it is worth noting that bodyweight values may be poor proxy measures for obesity status.

## Conclusions

This study of over one thousand Pugs documented steeply rising ownership of Pugs in England and provides important disorder information on the general population of Pugs. The most common disorders in Pugs were overweight/obesity, corneal disorder and otitis externa. The head-and-neck region was the most commonly affected body location. These results provide a framework to identify health priorities in Pugs and can contribute positively to reforms to improve health and welfare within the breed.

## Abbreviations

BOAS, brachycephalic obstructive airway syndrome; CI, confidence interval; EPR, electronic patient record; IQR, interquartile range

## Additional file

Additional file 1: Mapping systems that linked disorder terms between diagnosis-level precision, grouped-level precision and syndromic classifications. (XLSX 58 kb)
